# Ocular Surveillance During Off‐Label Oral Isotretinoin for Photoaging: A Review of Risks and Monitoring Recommendations

**DOI:** 10.1111/jocd.70401

**Published:** 2025-08-22

**Authors:** Fabiano Nadson Magacho‐Vieira, Gilmasa Daniele Rios Dias

**Affiliations:** ^1^ Magacho Institute for Health Education Fortaleza Ceará Brazil; ^2^ Department of Clinical, Aesthetic and Surgical Dermatology Batista Memorial Hospital Fortaleza Ceará Brazil

**Keywords:** adverse effects, dry eye syndrome, isotretinoin, photoaging, retinal diseases, retinoids, visual dysfunction

## Abstract

**Background:**

Low‐dose oral isotretinoin is increasingly used off‐label for photoaging, with evidence of clinical and histologic skin improvement.

**Objective:**

To review ocular risks associated with isotretinoin in aesthetic use and propose monitoring recommendations.

**Methods:**

A narrative review of clinical studies, observational data, and mechanistic reports addressing isotretinoin‐induced ocular effects was performed, integrating dermatologic and ophthalmologic perspectives.

**Results:**

Reported adverse effects include dry eye disease, meibomian gland dysfunction, and, rarely, retinal alterations. Risk may be cumulative in long‐term or repeated cosmetic regimens.

**Conclusion:**

A risk‐adapted monitoring approach, emphasizing patient selection, informed consent, and interdisciplinary care, is recommended to balance cosmetic benefits with ocular safety.

Oral isotretinoin, a synthetic retinoid derived from vitamin A, is widely used in managing severe acne. Recently, low‐dose isotretinoin has attracted attention as an off‐label intervention for photoaging, demonstrating positive clinical and histologic changes in skin appearance [1, 2, 3]. However, long‐term aesthetic use of isotretinoin requires careful evaluation of ocular risks and monitoring strategies to ensure patient safety. No formal ophthalmologic monitoring protocols are widely available for off‐label isotretinoin use. The authors propose a risk‐adapted approach based on their clinical experience and a comprehensive literature review, offering a practical and evidence‐based framework for case management.

## 1 Efficacy of Isotretinoin for Photoaging

Clinical studies have consistently demonstrated that oral isotretinoin administered at sub‐acne dosages (typically 10–20 mg, three times per week for 12 weeks) leads to significant improvements in dermal architecture [1, 2]. These include enhanced collagen synthesis and reduced solar elastosis, resulting in visibly firmer, more uniform skin. In a randomized comparative trial, Bagatin et al. [3] found that low‐dose oral isotretinoin offered histologic benefits comparable to those of topical tretinoin in patients with moderate to severe signs of photoaging.

Additional observational and prospective studies reinforce these findings, with patients frequently reporting subjective improvements in wrinkle depth, pigmentation, and overall skin texture [4, 5]. Other investigations underscore the durability of these clinical and histological outcomes, even following relatively brief treatment cycles, highlighting the drug's favorable tolerability profile when used at lower doses for aesthetic purposes [2, 5].

While these results offer a compelling rationale for aesthetic use, it is important to recognize that isotretinoin remains a systemic agent with known effects on epithelial structures. The growing enthusiasm for its application in cosmetic dermatology should be tempered with rigorous attention to patient selection, treatment duration, and monitoring of adverse events, especially when usage extends beyond dermatologic disease management to prevention of skin aging.

Commonly reported side effects include cheilitis, xerosis, mucocutaneous dryness, epistaxis, and, in some cases, ocular manifestations such as dry eye disease (DED). While generally expected to be less frequent and milder, these adverse effects may nonetheless occur within low‐dose regimens [6, 7]. Therefore, individualized monitoring remains essential, considering patient‐specific risk factors and clinical tolerance to the medication.

## 2 Ocular Adverse Effects

Recent data have underscored the high prevalence of ocular side effects in patients using isotretinoin. In a cross‐sectional study of 489 individuals, Abuallut et al. [8] found that blurred vision (75.9%), sore eyes (68.6%), and foreign body sensation (66.4%) were among the most frequently reported complaints. The severity of symptoms, as measured by the Ocular Surface Disease Index (OSDI), correlated positively with isotretinoin dosage and contact lens use. These findings are especially relevant in populations using isotretinoin for aesthetic indications, which often include middle‐aged adults, women, and individuals likely to wear contact lenses for either refractive correction or cosmetic purposes.

Isotretinoin‐induced meibomian gland dysfunction is a key pathophysiologic mechanism underlying these symptoms. The drug reduces sebaceous gland activity, including that of the meibomian glands, thereby impairing lipid secretion into the tear film and increasing evaporative tear loss [9]. Histological evidence also supports isotretinoin‐mediated apoptosis of meibomian gland epithelial cells and conjunctival goblet cell depletion, contributing to dry eye disease [8, 10]. These changes may be transient in some individuals but can result in chronic ocular surface disease, blepharoconjunctivitis, or intolerance to contact lens wear.

Beyond symptoms of surface irritation, isotretinoin use has been associated with structural and functional changes to the ocular surface. Decreased tear production, altered blinking patterns, and disruption of mucin‐producing cells can exacerbate ocular discomfort and interfere with visual acuity. These complications not only affect quality of life but may also limit the patient's ability to maintain visual activities such as reading, screen use, or night driving.

A large retrospective study by Zane et al. [11] documented ocular adverse effects beyond DED, including night blindness, photophobia, decreased dark adaptation, and in rare cases, optic neuritis—suggesting possible involvement of the optic nerve or retina. These findings highlight the need for closer clinical attention, especially in patients undergoing long‐term therapy for dermatologic indications. The underreporting of visual symptoms in clinical trials further emphasizes the need for proactive inquiry by clinicians.

Retinoids play a central role in the visual cycle, particularly in the regeneration of 11‐cis‐retinal, the chromophore essential for phototransduction. Experimental data from animal models have demonstrated that disruption in retinoid signaling can lead to retinal degeneration and photoreceptor dysfunction [12]. Mutations in genes involved in the retinoid cycle, such as RPE65, have been directly linked to retinal dystrophies such as Leber congenital amaurosis and retinitis pigmentosa. These parallels raise concern about the long‐term modulation of retinoid pathways in humans exposed to isotretinoin.

Although definitive evidence of isotretinoin‐induced retinal toxicity in humans remains limited, several reports describe alterations in color vision, nyctalopia, and electrophysiologic changes suggestive of retinal involvement [11, 12]. These effects may be subtle or reversible, but their potential to accumulate over time with extended isotretinoin exposure raises concern, particularly in off‐label contexts where the duration of therapy is not standardized.

In this context, the expanding use of oral isotretinoin for cosmetic purposes—often involving repeated cycles—may elevate the risk of cumulative retinal stress, especially in individuals with undiagnosed or subclinical retinal disorders [12, 13, 14]. This risk is amplified in aging populations, which already face an increased prevalence of retinal pathology such as age‐related macular degeneration and diabetic retinopathy [12].

## 3 Clinical Recommendations

In aesthetic indications, where isotretinoin is used at lower doses, the risk of clinically significant ocular complications is presumed to be low, and routine ophthalmologic monitoring may be more applicable to high‐dose, long‐term regimens. Nevertheless, this reduced risk does not preclude the need for ophthalmologic vigilance. Although no formal ophthalmologic monitoring protocols have been established for the off‐label use of isotretinoin in aesthetic indications, with most current recommendations being adapted from acne treatment protocols [13, 14, 15], expert reviews and consensus statements advocate for a risk‐adapted approach [1, 2, 3, 15].

In contrast to acne therapy, the use of isotretinoin for photoaging is typically elective and may extend over longer periods. This might increase the cumulative dose and potential for adverse events. As such, clinicians should consider a risk‐based approach to ophthalmologic monitoring, particularly in predisposed individuals. Table 1 provides an overview of the relevant risk conditions and the clinical reasoning for their inclusion.

**TABLE 1:** Clinical scenarios associated with increased ophthalmologic risk during oral isotretinoin therapy for photoaging. Risk factors and their justifications are supported by published data correlating isotretinoin use with ocular surface compromise and, in select cases, retinal involvement. Reference numbers correspond to sources cited in the main text.

Ophthalmologic risk factors	Justification for monitoring
Preexisting dry eye or ocular surface disease	Aggravation due to isotretinoin‐induced meibomian gland dysfunction and tear instability [8, 11].
Regular contact lens use	Increased evaporative stress, significantly associated with higher OSDI scores and dry eye symptoms [8, 9].
Prior refractive surgery	Altered corneal sensitivity and tear dynamics increase vulnerability to dry eye disease [8, 10].
Existing retinal or optic nerve conditions	Potential exacerbation due to disrupted retinoid metabolism affecting retinal integrity and function [11, 12].
Older age or systemic diseases (e.g., diabetes)	Higher baseline risk of retinal and ocular surface disease; age‐related tear film instability [5, 12].
Occupations with prolonged screen exposure	Increased blink suppression and evaporative tear loss may exacerbate dry eye symptoms in isotretinoin users [8].

Baseline assessment may be appropriate in high‐risk patients, while follow‐up should be guided by symptoms [1, 2]. Those may include slit‐lamp examination, tear break‐up time, Schirmer testing, and evaluation of meibomian gland morphology [8, 13, 14]. Subsequent follow‐up should be symptom‐driven, with early use of ocular lubricants, topical anti‐inflammatories, or punctal plugs as indicated [8, 13]. Patients should be explicitly counseled to report new or worsening ocular symptoms, including photophobia, blurred vision, and reduced night vision [8, 13, 14].

Routine fundoscopic or imaging studies are not currently recommended for all patients. However, referral to ophthalmology should be pursued when visual disturbances are persistent, progressive, or associated with functional impairment. Electroretinography or optical coherence tomography may be warranted in select cases [13].

Additionally, in patients presenting with ocular symptoms during isotretinoin therapy, clinicians should also consider differential diagnoses beyond drug‐induced effects. Conditions such as Demodex blepharitis, ocular rosacea, conjunctivitis, and chronic meibomian gland dysfunction may produce overlapping symptoms, including foreign body sensation, ocular dryness, and eyelid inflammation. A careful ophthalmologic evaluation is essential to avoid inappropriate attribution of ocular symptoms to isotretinoin, particularly when symptoms persist or appear disproportionate to the systemic dose administered.

**FIGURE 1:**
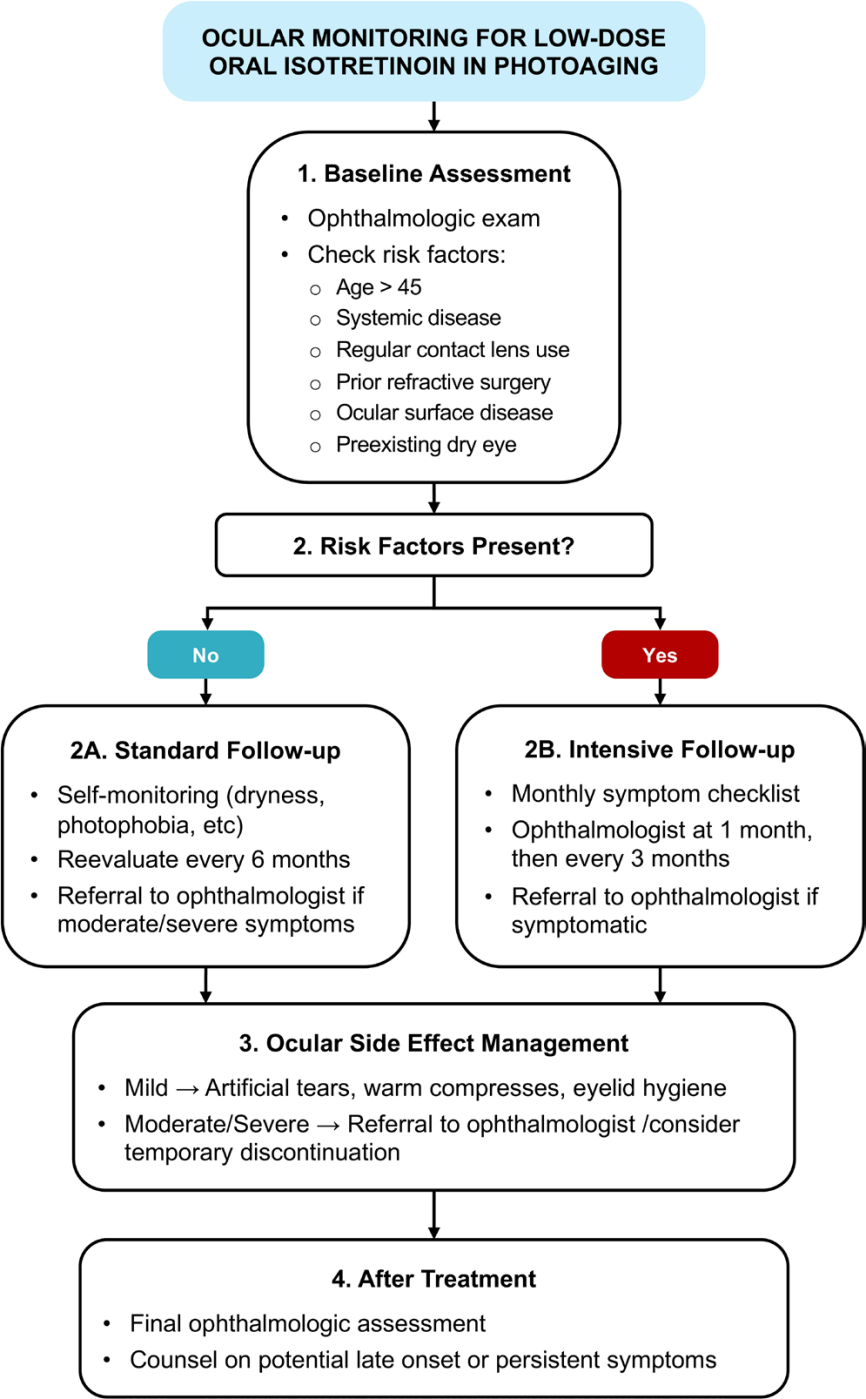
Flowchart outlining a risk‐based approach to ophthalmologic monitoring in patients treated with low‐dose oral isotretinoin for photoaging. The algorithm is structured into four stages: Baseline assessment, follow‐up during treatment, management of ocular side effects, and post‐treatment evaluation. Patient stratification is based on predefined risk factors, guiding clinicians toward standard or intensive monitoring protocols. Arrows direct decision‐making through each phase, emphasizing symptom‐based follow‐up and early intervention when necessary.

Beyond direct monitoring, interdisciplinary collaboration between dermatologists and ophthalmologists is strongly encouraged. Dermatologists should be aware of the ocular risks and communicate openly with patients and their eye care providers to ensure that systemic treatments do not compromise visual health. This is particularly important given the elective nature of cosmetic indications, where the threshold for acceptable risk is lower. Figure 1 illustrates a stepwise approach to ophthalmologic monitoring for patients treated with low‐dose oral isotretinoin.

## 4 Conclusion

Low‐dose oral isotretinoin has emerged as a promising off‐label option for the treatment of photoaging, with growing evidence supporting its clinical and histological benefits [3, 10]. Despite its expanding role in cosmetic dermatology, ocular outcomes remain underexplored in this context.

Current data do not demonstrate clear superiority over topical retinoids, and comparative studies with procedural therapies are lacking, limiting a clear risk–benefit assessment in aesthetic settings [10]. Given its systemic nature and potential for ocular adverse effects, caution is warranted, particularly as its use expands beyond short‐term acne treatment into long‐term preventive dermatology.

These considerations support a structured, risk‐adapted monitoring strategy, especially in patients with predisposing factors such as older age, systemic diseases (e.g., diabetes), contact lens use, prior refractive surgery, or baseline ocular surface disease. Baseline ophthalmologic evaluation and symptom‐driven follow‐up should be incorporated into clinical practice until more definitive guidelines are established. Interdisciplinary collaboration will be key to balancing therapeutic efficacy with long‐term ocular safety.

## Author Contributions

The authors confirm contribution to this article as follows. Conception, design, and draft preparation: Fabiano Nadson Magacho‐Vieira and Gilmasa Daniele Rios Dias. All authors reviewed, approved, and agreed to be accountable for all aspects of the final version of this article.

## Ethics Statement

The authors have nothing to report.

## Consent

The authors have nothing to report.

## Conflicts of Interest

Fabiano Nadson Magacho‐Vieira serves as medical director for Derma Dream Corporation. Gilmasa Daniele Rios Dias declares no conflicts of interest.

## Data Availability

Data sharing is not applicable to this article as no new data were created or analyzed in this study.
